# Effect of IL-10-producing B cells in peripheral blood and tumor tissue on gastric cancer

**DOI:** 10.1186/s12964-023-01174-5

**Published:** 2023-11-09

**Authors:** Yoon Ju Jung, Jin Seok Woo, Sun-Hee Hwang, SeungCheon Yang, So Jung Kim, JooYeon Jhun, Seung Yoon Lee, Kun Hee Lee, Mi-La Cho, Kyo Young Song

**Affiliations:** 1 Division of Gastrointestinal Surgery, Department of Surgery, Yeouido St. Mary’s Hospital, College of Medicine, The Catholic University of Korea, Seoul, 07345 Korea; 2 Rheumatism Research Center, Catholic Research Institute of Medical Science, College of Medicine, The Catholic University of Korea, Seoul, 06591 Korea; 3 Lab of Translational ImmunoMedicine, Catholic Research Institute of Medical Science, College of Medicine, The Catholic University of Korea, Seoul, 06591 Korea; 4 Division of Gastrointestinal Surgery, Department of Surgery, Seoul St. Mary’s Hospital, College of Medicine, The Catholic University of Korea, Seoul, 06591 Korea; 5 Department of Biomedicine & Health Sciences, College of Medicine, The Catholic University of Korea, Seoul, 06591 Korea; 6 Department of Medical Life Sciences, College of Medicine, The Catholic University of Korea, Seoul, 06591 Korea

**Keywords:** Stomach neoplasm, Carcinogenesis, Prognosis, IL-10-producing B (B10) cells, Interleukin-10 (IL-10)

## Abstract

**Background:**

Interleukin (IL)-10-producing B (B10) cells are generated in response to signals from the tumor microenvironment and promote tumor growth by interacting with B10 cells. We investigated the distributions of immune cells in peripheral blood and tumor tissue samples from patients with gastric cancer (GC).

**Methods:**

Patients with GC who underwent radical gastrectomy in Seoul St. Mary’s Hospital between August 2020 and May 2021 were enrolled in this study. Forty-two samples of peripheral blood were collected, and a pair of gastric mucosal samples (normal and cancerous mucosa; did not influence tumor diagnosis or staging) was collected from each patient after surgery. B10 cells in peripheral blood and cancer mucosa samples were investigated by flow cytometry and immunofluorescence. AGS cells, gastric cancer cell line, were cultured with IL-10 and measured cell death and cytokine secretion. Also, AGS cells were co-cultured with CD19 + B cells and measured cytokine secretion.

**Results:**

The population of B10 cells was significantly larger in the blood of patients with GC compared with controls. In confocal images of gastric mucosal tissues, cancerous mucosa contained more B10 cells than normal mucosa. The population of B10 cells in cancerous mucosa increased with cancer stage. When AGS cells were cultured under cell-death conditions, cellular necrosis was significantly decreased, and proliferation was increased, for 1 day after IL-10 stimulation. Tumor necrosis factor (TNF)-α, IL-8, IL-1β, and vascular endothelial growth factor secretion by cancer cells was significantly increased by coculture of AGS cells with GC-derived CD19^+^ B cells.

**Conclusions:**

B cells may be one of the populations that promote carcinogenesis by inducing the production of inflammatory mediators, such as IL-10, in GC. Targeting B10 cells activity could improve the outcomes of antitumor immunotherapy.

Video Abstract

**Supplementary Information:**

The online version contains supplementary material available at 10.1186/s12964-023-01174-5.

## Background

Gastric cancer (GC) has a high mortality rate worldwide, and early diagnosis by screening increases the survival rate [1]. In western societies, where screening endoscopy is not performed, median survival does not exceed 2 years when the disease is classified as inoperable after symptom onset, or when GC recurs after treatment with curative intent [2]. Immune checkpoint inhibitors targeting programmed cell death-1 receptor or its ligand have less efficacy than in other cancers [3].

Tumor cells in patients with GC are highly invasive and metastatic as a result of their immune-escape mechanisms, defects in the functions of antitumor killer cells, such as cytotoxic T cells and natural killer cells, the presence of immunosuppressive cells and their inhibitory cytokines, and the expression of inhibitory molecules on the surface of tumor cells [4, 5]. B cells, especially tumor-infiltrating B cell subsets, may exert direct or indirect effects on GC cells. B10 cells may have an immunosuppressive effect on GC cells by blocking the differentiation of proinflammatory lymphocytes, and producing cytokines such as interleukin (IL)-10 and tumor growth factor (TGF)-β [6–9]. B10 cells induce the differentiation of immunosuppressive T cells [10, 11]. Tumor-infiltrative B cell subsets are independent good prognostic factors in patients with GC, and greater infiltration of the tumor environment by B10 cells results in a worse prognosis [7].

IL-10 production by B cell may be associated with the inhibition by regulatory T cells of IL-2, IL-5, and tumor necrosis factor (TNF)-α production, or interactions with antigen-presenting cells (APCs) that decrease major histocompatibility complex antigen expression [8]. Therefore, IL-10-producing B cells suppress antitumor immunity. Indeed, patients with GC showed higher infiltration of B10 cells in tumor tissue and peripheral blood, which increased with tumor stage [12].

We evaluated the populations of B10 cells in peripheral blood and tumor tissue from patients with GC.

## Methods

### Study population

Patients with GC preoperatively confirmed as adenocarcinoma by endoscopic biopsy were enrolled in this study. The patients underwent conventional radical gastrectomy with curative intent in Seoul St. Mary’s Hospital August 2020 to May 2021, according to the Korean Gastric Cancer Treatment Guidelines. Patients with early GC underwent D1 + lymph node (LN) dissection, and patients with locally advanced cancer underwent D2 or D2 + LN dissection. Forty-two peripheral blood and gastric mucosal tissue samples were collected, and a pair of gastric mucosal samples (normal and cancerous mucosa; did not influence tumor diagnosis and staging) was obtained from each patient after surgery. Blood samples were collected from five patients 1–3 months after surgery and compared with preoperative samples. Preoperative peripheral blood samples from the patients with GC were compared with those from 13 healthy controls. Pathologic stage of GC was classified according to the 8th American Joint Committee on Cancer criteria. This study was approved by the Institutional Review Board of the College of Medicine, Catholic University of Korea (KC20TISI0985). Patient records were anonymized and de-identified before analysis.

### Intracellular staining and flow cytometry

Human peripheral blood mononuclear cells were isolated from blood samples [13], and stimulated with 25 ng/mL phorbol myristate acetate and 250 ng/mL ionomycin (Sigma-Aldrich, St. Louis, MO) in the presence of GolgiStop (BD Biosciences, San Jose, CA) for 4 h. Surface staining was performed with surface fluorescein isothiocyanate (FITC)-conjugated anti-CD19, phycoerythrin-Cy7-conjugated anti-CD24, peridinin chlorophyll protein-conjugated anti-CD38, and Brilliant Violet™ 421-conjugated anti-CD138 (BD Pharmingen, Franklin Lakes, NJ) antibodies. Surface-labeled cells were permeabilized using Cytofix/Cytoperm solution (BD Pharmingen, Franklin Lakes, NJ), and subjected to intracellular staining using an allophycocyanin (APC)-conjugated anti-IL-10 antibody. Samples were analyzed using the FACS Calibur (BD Pharmingen) fluorescence-activated cell sorting (FACS) instrument, and data were analyzed with FlowJo software (Tree Star, Ashland, OR). The antibodies used are listed in Table 1.

**Table 1:** List of antibodies used in this study

Target	Source	Identifier	Application
FITC-CD19	BD	555,412	Flow Cytometry
PE-Cy7-CD24	BD	561,646	Flow Cytometry
PerCP5.5-CD38	BD	551,400	Flow Cytometry
BV421-CD138	BD	562,935	Flow Cytometry
APC-IL-10	Biolegend	501,410	Flow Cytometry
FITC-CD19	BD	555,412	Immunofluorescence
APC-IL-10	Biolegend	506,807	Immunofluorescence

### Confocal microscopy

Mucosal tissues of patients with GC were fixed in 10% formalin after decalcification using Decalcifying Solution-Lite (Sigma), and embedded in paraffin. Paraffin-embedded sections were incubated at 4 °C with APC-conjugated anti-IL-10 (Biolegend, San Diego, CA), and FITC-conjugated anti-CD19 (BD Pharmingen, Franklin Lakes, NJ) antibodies. Nuclei were stained with 4,’6-diamidino-2-phenylindole (DAPI; Invitrogen, Carlsbad, CA). Images were obtained using an LSM 700 confocal microscope (Zeiss, Oberkochen, Germany) at 200 × magnification. The antibodies used are listed in Table 1

### Annexin V-FITC/PI staining

Cellular apoptosis and necrosis were assayed using an Apoptosis Detection Kit (BioVision, Milipitas, CA). AGS cells were seeded in 24-well culture plates at 8 × 10^4^ per well and stimulated with an anti-IL-10 (40 ng/mL) antibody for 24 h. The cells were washed twice with phosphate-buffered saline (PBS), resuspended in 500 µL of binding buffer, and incubated with 5 µL of annexin V-FITC and 5 µL of propidium iodide (PI) for 5 min in the dark. The apoptosis and necrosis rates were calculated by determining the percentages of annexin V^+^/PI^+^ and annexin V^−^/PI^+^ cells, respectively. Samples were analyzed using the FACS Calibur instrument (BD Pharmingen), and data were analyzed with FlowJo software.

### Cell proliferation assay

Cell proliferation was assayed using the Cell Counting Kit-8 (CCK-8; Dojindo Molecular Technologies, MD). AGS cells were seeded in 96-well culture plates at 2 × 10^4^ per well and stimulated with an anti-IL-10 (20 ng/mL) antibody for 48 h. Next, 20 μL of CCK-8 solution was added to each well, and the plates were incubated for 1–3 h at 37 °C with 5% CO_2_. The absorbance at 450 nm was measured using a microplate reader (Molecular Devices, Sunnyvale, CA).

### Enzyme-linked immunosorbent assay

IL-8, IL-1β, TNF-α, and vascular endothelial growth factor (VEGF) levels in culture supernatants were determined by sandwich enzyme-linked immunosorbent assay (ELISA) (DuoSet; R&D Systems, Lille, France). Horseradish peroxidase-conjugated streptavidin was used for color development. Absorbance at 450 nm was measured using an ELISA microplate reader (Molecular Devices).

### Cell culture

AGS cells were purchased from the Korean Cell Line Bank. The cells were cultured in Roswell Park Memorial Institute-1640 medium with 10% heat-inactivated fetal bovine serum and antibiotics at 37 °C in 5% (v/v) CO_2_. Cells were stimulated with an anti-lipopolysaccharide (LPS) (100 ng/mL), -TNF-α (10 ng/mL), or -IL-10 (20 or 40 ng/mL) antibody for 24 or 48 h. CD19^+^ B cells were isolated from peripheral blood mononuclear cells of patients with GC using CD19 MicroBeads (130–050-301, Miltenyi Biotec) following the manufacture’s instruction and cocultured with AGS cells (10:1 ratio) for 72 h. Recombinant human anti-IL-10 and -TNF-α antibodies were purchased from R&D Systems (Minneapolis, MN) and LPS was obtained from Sigma-Aldrich.

### Ethics approval and consent to participate

This study was approved by the Institutional Review Board of the College of Medicine, Catholic University of Korea (KC20TISI0985) in accordance with the Declaration of Helsinki and Good Clinical Practice guidelines. All procedures were in accordance with the ethical standards of the responsible committee on human experimentation (institutional and national), and with the Helsinki Declaration of 1964 and later versions. Informed consent or an appropriate substitute was obtained from all patients included in the study.

### Statistical analysis

Data are means ± standard errors of the mean. Statistical analysis was performed using Prism software for Windows (ver. 5; GraphPad Software Inc., San Diego, CA). Normally distributed continuous data were analyzed by parametric Student’s *t-*test. Group means were compared by one-way analysis of variance. *P* < 0.05 was deemed to indicate statistical significance.

## Results

### Patient characteristics

The clinicopathological characteristics of the participants are shown in Table 2. The mean age of the patients with GC was 64.61 ± 10.41 years. Among the 42 patients with GC, 30 (71.4%) were male and the mean body mass index was 23.77 ± 3.58. Participants in healthy control group were younger, and had more female patients (*P* < 0.05, 0.031, respectively). Smoking history and comorbidities were also different between two groups (Table 2). After radical gastrectomy, among GC patients, 20 (47.6%) were diagnosed with early GC, and 22 (52.3%) with advanced GC. Twenty-three patients with GC (54.7%) had no LN metastasis.

**Table 2:** Clinicopathological characteristics of the patients with gastric cancer and healthy control

		**Patients with gastric cancer *N* = 42**	**Healthy Control** ***N* = 13**	***P* value**
**Age, mean (SD)**		64.61 (± 10.41)	45.77 (± 19.12)	< 0.001
**Sex (M:F ratio)**	**M**	30 (71.4)	5 (38.5)	0.031
	**F**	12 (28.6)	8 (61.5)	
**BMI, mean (SD)**		23.77 (± 3.58)	25.39 (± 6.00)	0.346
**ECOG**	**0**	34 (80.9)	11 (84.6)	0.765
	**1**	8 (19.0)	2 (15.4)	
	**2**	0		
**Smoking (%)**	**Never**	16 (38.0)	12 (92.3)	0.003
	**Quit**	11 (26.1)	0	
	**Active**	15 (35.7)	1 (7.7)	
**Alcohol (%)**	**Never**	28 (66.6)	8 (61.5)	0.357
	**Social**	10 (23.8)	5 (38.5)	
	**Heavy**	4 (9.5)	0	
**Comorbidities**	**Hypertension**	19 (45.2)	1 (7.7)	0.018
	**DM**	7 (16.6)	2 (15.4)	
	**Cardiovascular**	3 (7.1)	0	
	**Pulmonary**	2 (4.7)	0	
**pT stage**	**T1**	20 (47.6)		
	**T2-4**	22 (52.3)		
**pN stage**	**N0**	23 (54.7)		
	**N1-3**	19 (45.2)		
**pM stage**	**M0**	41 (97.6)		
	**M1**	1 (2.3)		
**pStage 8**^**th**^	**I**	23 (54.7)		
	**II**	8 (19.0)		
	**III**	10 (23.8)		
	**IV**	1 (2.3)		
**Differentiation**	**Differentiated**	13 (30.9)		
	**Undifferentiated**	29 (69.0)		
**Lauren**	**Intestinal**	13 (30.9)		
	**Diffuse/Mixed**	26 (61.9)		
**Lymphatic invasion**	**Negative**	24 (57.1)		
	**Positive**	18 (42.8)		
**Vascular invasion**	**Negative**	38 (90.1)		
	**Positive**	4 (9.5)		
**Neural invasion**	**Negative**	29 (69.0)		
	**Positive**	13 (30.9)		

Data are numbers (percentages) or means (± SD). The chi-squared test was used to test for between-group differences in categorical variables; *P* < 0.05 was deemed indicative of statistical significance

*BMI* Body mass index, *ECOG* Eastern Cooperative Oncology Group score

### Analysis of peripheral blood samples

**Fig. 1:**
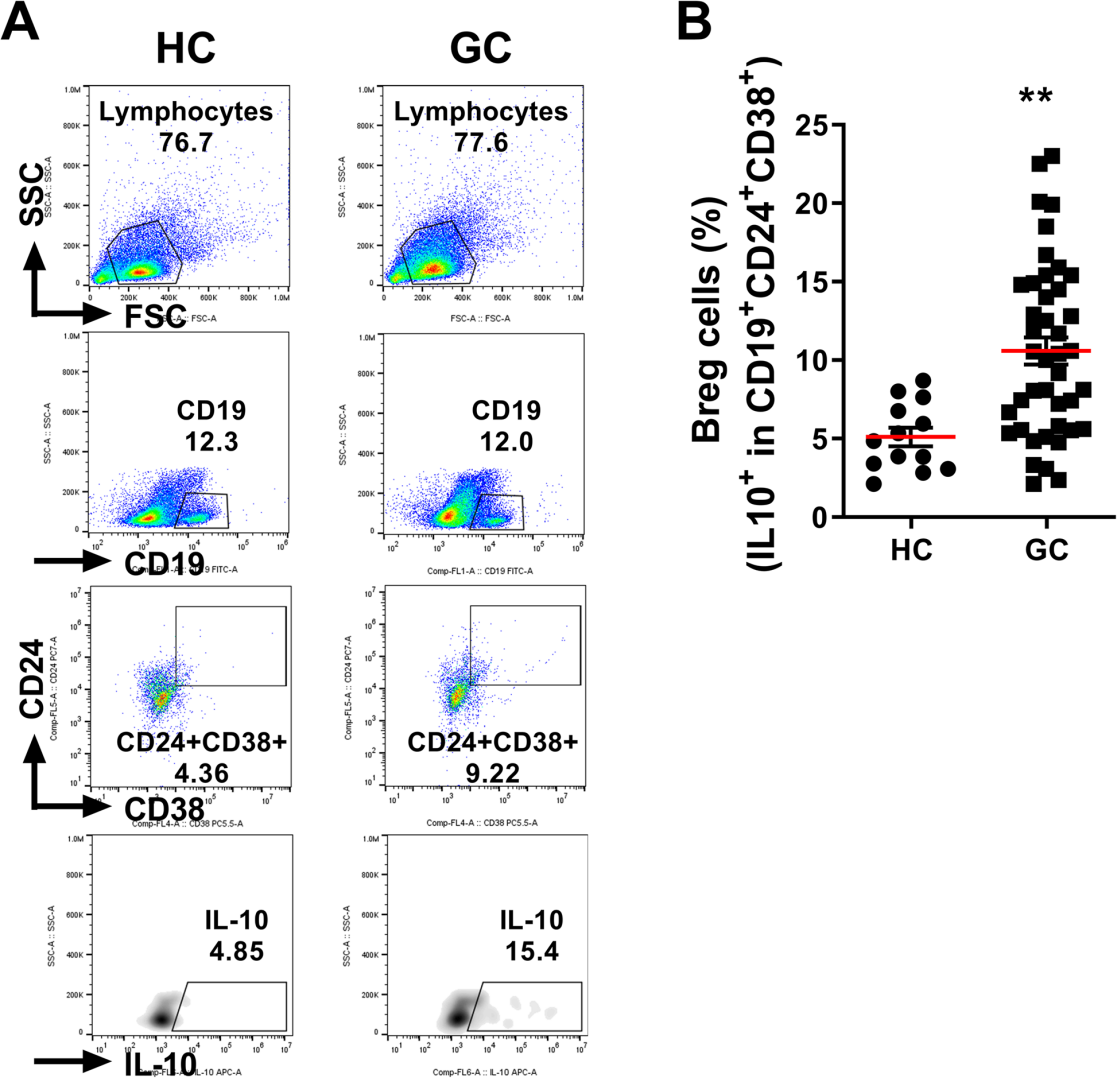
Increased B10 population in PBMCs of patients with GC. PBMCs of healthy controls (HC) and patients with GC were stimulated with PMA and ionomycin for 4 h, and GolgiStop for the final 2 h. **A** Representative FACS plots with B10 cells gating (IL-10^+^ in CD19^+^CD24^+^CD38.^+^). **B** Mean percentage of B10 in PBMCs of HCs (*N* = 13) and patients with GC (*N* = 42). Means ± SEM (**P < 0.01)

**Fig. 2:**
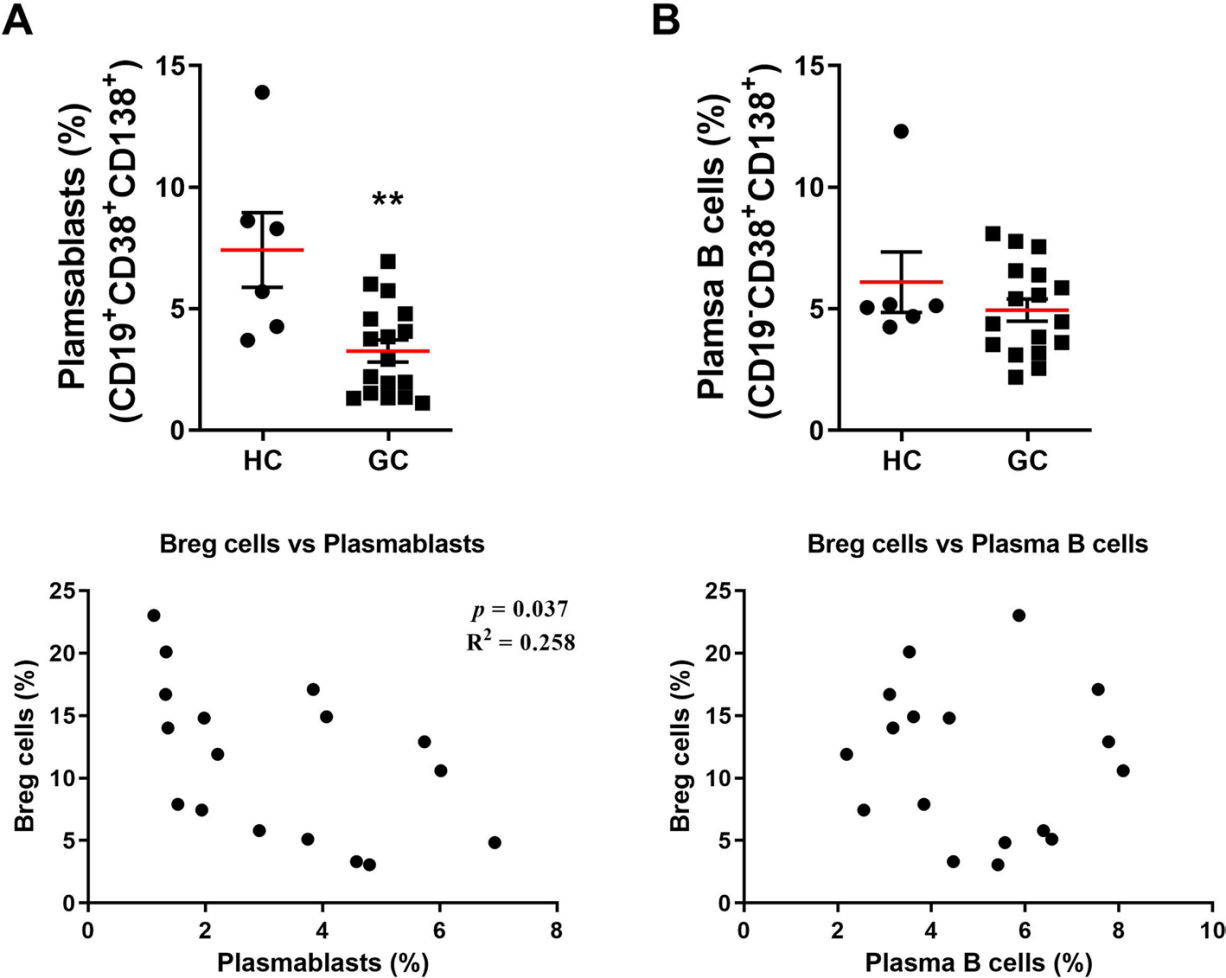
B-Cell subgroups in patients with GC. **A** Top, mean percentages of plasmablasts (CD19^+^CD38^+^CD138^+^) in PBMCs from HCs (*N* = 6) and patients with GC (*N* = 17). Bottom, correlation between B10 cells and plasmablasts. **B** Top, mean percentages of plasma B cells (CD19^−^CD38^+^CD138.^+^) in PBMCs of HCs (*N* = 6) and patients with GC (*N* = 17). Bottom, correlation between B10 cells and plasma B cells. Means ± SEM (**P < 0.01)

**Fig. 3:**
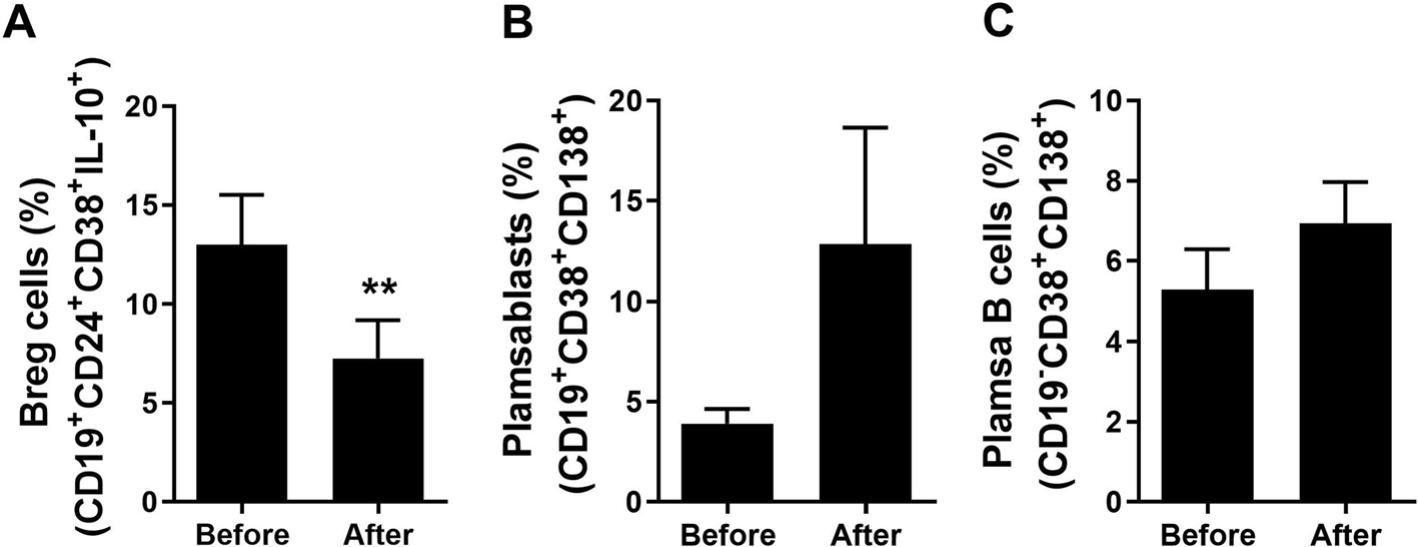
Improved immune suppression in patients with GC after surgery. PBMCs obtained from patients with GC before (*N* = 5) and after (*N* = 5) surgery were stimulated with PMA and ionomycin for 4 h, and GolgiStop for the last 2 h. Mean percentages of (**A**) B10 cells, (**B**) plasmablasts, and (**C**) plasma B cells are shown. Means ± SEM (**P < 0.01)

FACS analysis showed that the population of B10 cells (CD19^+^CD24^+^CD38^+^IL-10^+^) was significantly larger in the blood of patients with GC compared with controls (14.5 and 4.85, respectively, *P* < 0.01, Fig. 1). The population of plasmablasts (CD19^+^CD38^+^CD138^+^) was significantly smaller in PBMCs of patients with GC (*P* < 0.01, Fig. 2A), and was negatively correlated with B10 cells (*P* < 0.05, Fig. 2A). The population of plasma cells (CD19^−^CD38^+^CD138^+^) was non-significantly smaller in the patients with GC, but there was no quantitative correlation (Fig. 2B). PBMC staining showed that the B10 population decreased, and while the plasmablast and plasma cell populations showed an increasing trend, after radical gastrectomy (Fig. 3).

### Analysis of gastric mucosal tissue

**Fig. 4:**
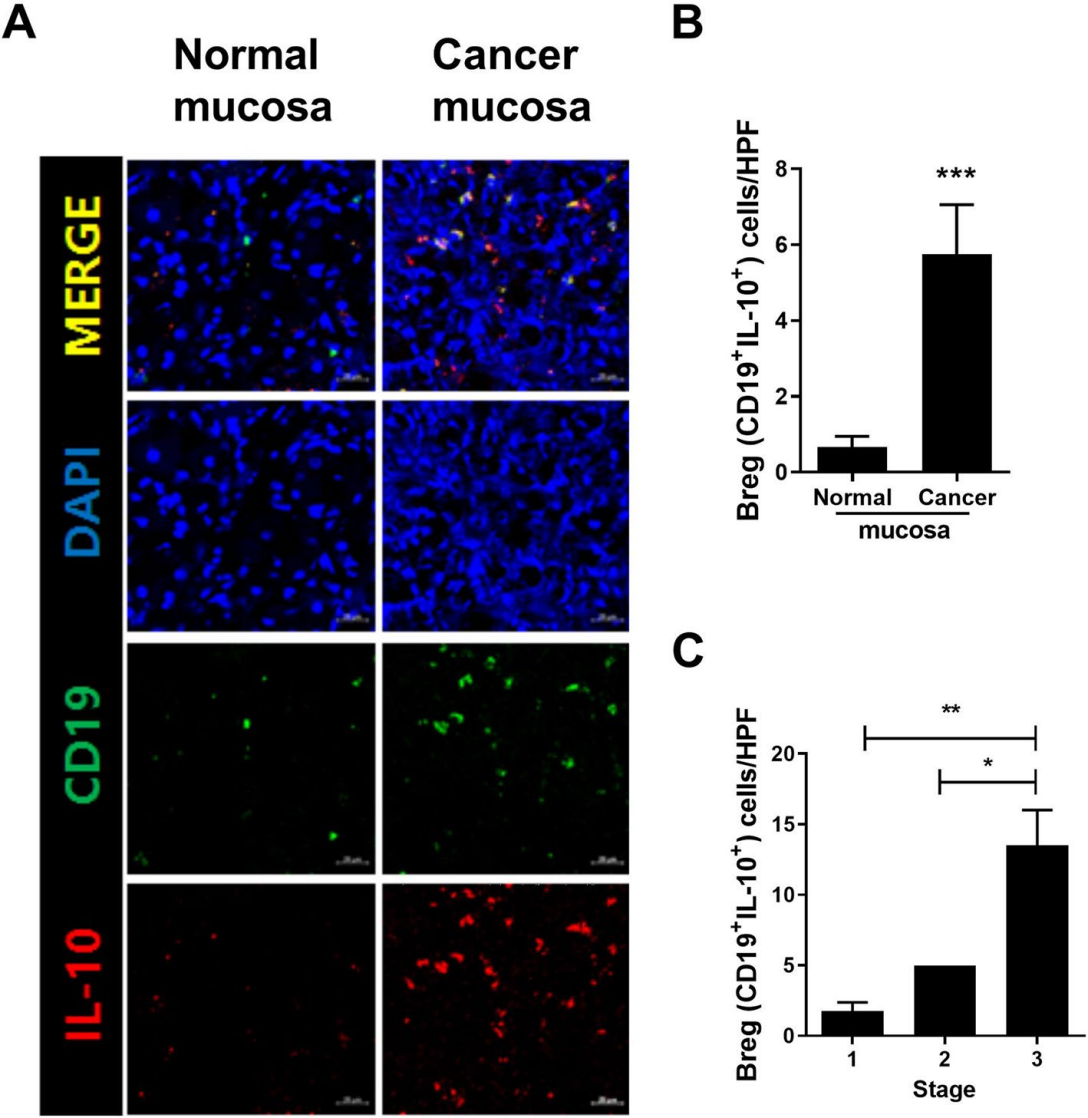
Increased B10 cells in GC mucosa. Normal and cancerous mucosae of patients with GC were stained with PE-IL-10, FITC-CD19, and DAPI. **A** Representative confocal images of B10 cells (CD19^+^IL-10.^+^) in normal (*N* = 12) and cancerous (*N* = 12) mucosae. Scale bar 20 μm. **B** Mean number of B10 cells per high-powered field (HPF) in normal and cancerous mucosae. **C** Mean number of B10 cells per HPF in cancer tissue of different stages. Means ± SEM (**P < 0.01)

Confocal imaging showed that cancerous mucosa contained a larger B10 population than normal mucosa (5.75 and 0.67, *P* < 0.001, Fig. 4A and B). The B10 population in cancer tissue increased with increasing tumor stage (stage I < II < III, *P* < 0.05, *P* < 0.01, Fig. 4C).

### Analysis of GC cells

**Fig. 5:**
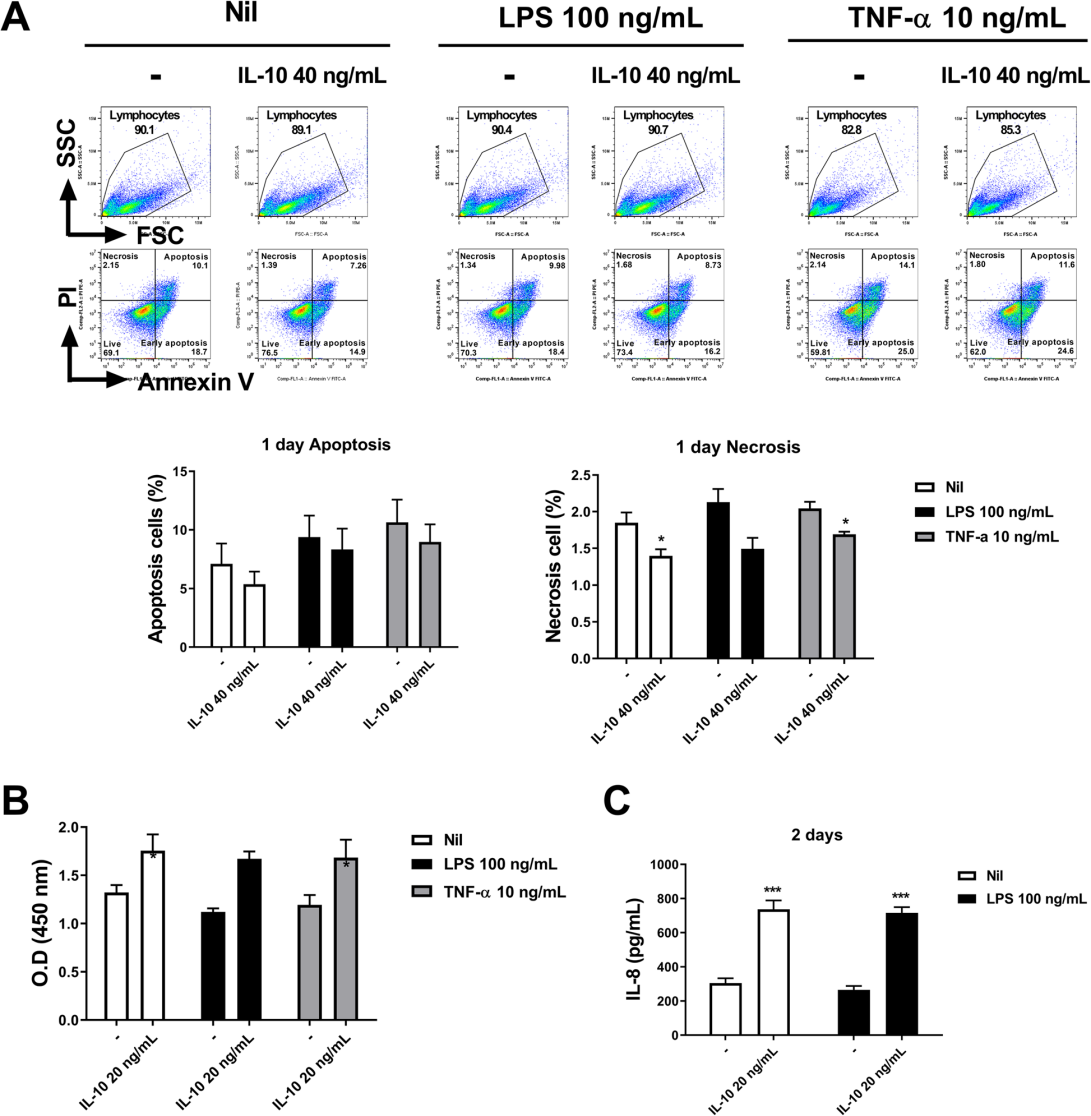
Effect of IL-10 on cancer cells. AGS GC cells were cultured in the absence or presence of IL-10 (10 ng/mL) with LPS (100 ng/mL) or TNF-α (10 ng/mL) for 24 h (for Annexin V and PI staining) and 48 h (for CCK-8 assay and IL-8 ELISA). **A** On day 1, cells were stained with Annexin V and PI. Representative FACS plots show the populations of apoptotic and necrotic cells. Bar graphs show the mean percentages of apoptotic (left) and necrotic (right) cells. **B** Mean OD values by CCK-8 assay. **C**. Mean IL-8 levels in culture supernatants. Means ± SEM (*P < 0.05, ***P < 0.005)

**Fig. 6:**
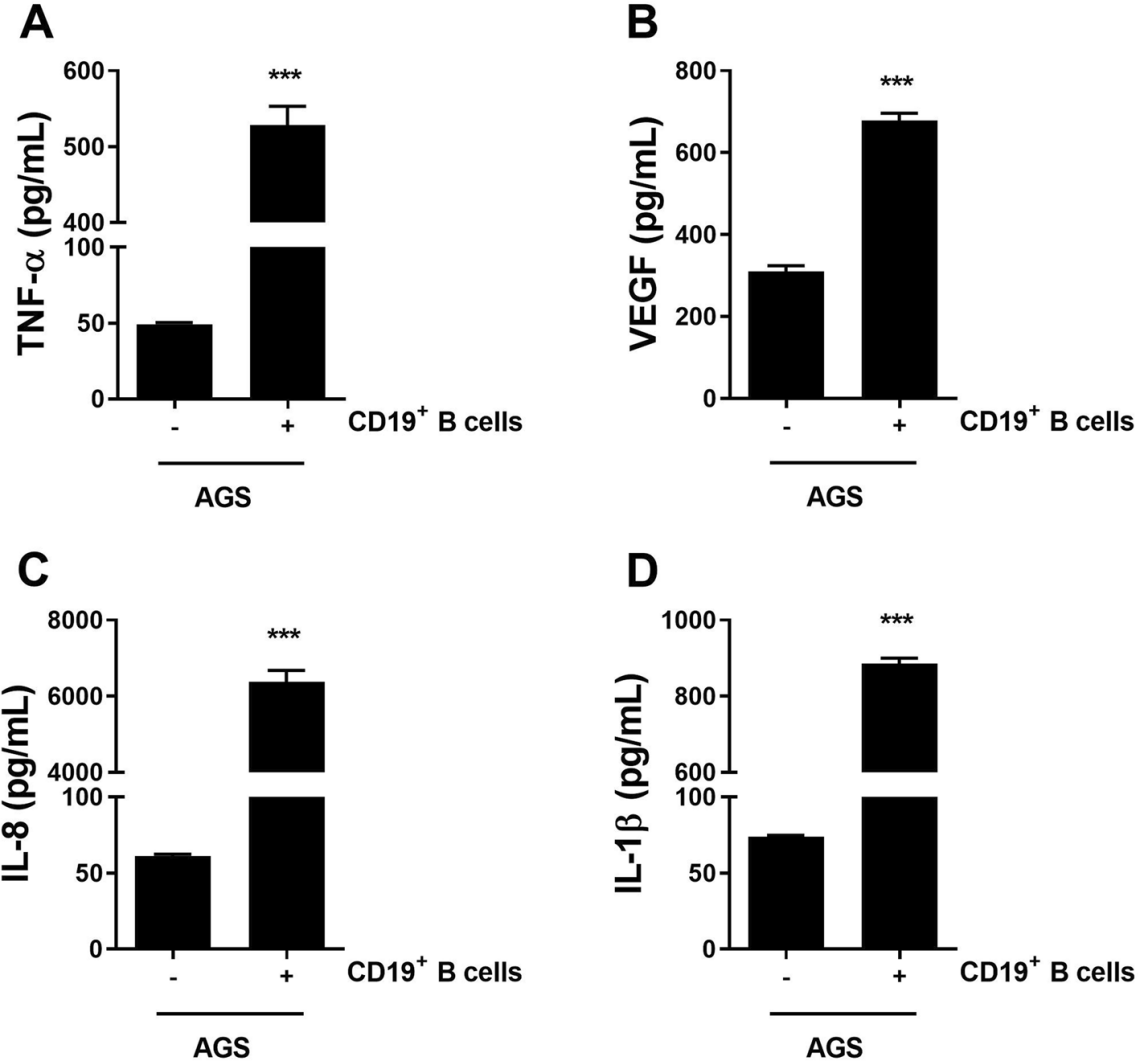
Effects of B cells from patients with GC on cytokine production. AGS cells were cocultured with B cells from patients with GC for 24 h. Levels of (**A**) TNF-α (top and left), **B** VEGF (top and right), **C**. IL-8 (bottom and left), and (**D**) IL-1β (bottom and right) are shown. Means ± SEM (***P < 0.005)

We cultured AGS cells with IL-10 in the presence of LPS and TNF-α. GC cell apoptosis and necrosis decreased for 1 day after IL-10 stimulation, and the number of necrotic cells differed significantly between TNF-α stimulation and non-stimulation conditions (both *P* < 0.05, Fig. 5A). Under the same conditions, AGS cell proliferation was significantly increased by IL-10 (*P* < 0.05, *P* < 0.001, Fig. 5B). In AGS cells cultured in the presence or absence of LPS, IL-8 secretion was significantly increased after 2 days of IL-10 treatment (*P* < 0.001, Fig. 5C). Interestingly, TNF-α, VEGF, IL-8, and IL-1β secretion by GC-derived CD19^+^ B cells was significantly increased by coculture with AGS cells (*P* < 0.001, Fig. 6).

## Discussion

GC has a high mortality rate worldwide. Except for East Asia, where endoscopic screening is performed, > 50% of patients with GC experience recurrence despite treatment [14, 15]. The mean survival time after recurrence of GC is ≤ 20 months [15]. Except for cytotoxic anticancer drugs, evidence of the benefits of alternative treatment options is ambiguous. Although conversion to surgery is expected to benefit patients who respond to anticancer drugs, evidence is lacking [16]. Targeted agents and immune checkpoint inhibitors are suitable for a small subset of patients, but are of little benefit [17–19]. Therefore, novel means of improving the prognosis of patients with stage 4 or recurrent GC are needed.

Cancer treatment aims to restore, maintain, and/or enhance the immune system to promote the detection and removal of cancer cells. Previously, treatment focused on activating innate immune cells, such as natural killer and dendritic cells, but was insufficiently efficacious [9, 18, 19]. Efforts have been made to enhance the action of T and B cells targeting tumor cells, and immune checkpoint inhibitors targeting programmed cell-death protein 1 (PD1), programmed death ligand 1 (PD-L1), or cytotoxic T-lymphocyte-associated protein 4 may be effective in patients with GC for palliative purposes [18]. An alternative is to maximize the antitumor effect using chimeric antigen receptor T cells, but further work is required before clinical application.

In this study, the B10 cells population was significantly larger in peripheral blood from patients with GC compared with controls (Fig. 1). IL-10 induces immunosuppression via signal transducer and activator of transcription (STAT) 3-mediated signal transduction, which involves tyrosine kinase phosphorylation and activation of STAT transcription factors [11]. IL-10 prevents the maturation of dendritic cells and inhibits the proliferation and cytokine production of CD4^+^ T cells [20].

In prostate or breast cancer, IL-10^+^CD19^+^ cells suppress effector immune cells in the tumor microenvironment by producing IL-10, and promote cancer metastasis by inducing regulatory T cells (Tregs) and releasing anti-inflammatory mediators [21]. IL-10^+^CD19^+^CD24^+^CD38^+^PD-L1^+^ B10 cells are associated with Treg proliferation and differentiation, and inhibit the antitumor responses of PD-1^hi^ effector T cells. Moreover, B10 cells control the expression of genes involved in the transition of B cells to plasmablasts, resulting in breast cancer invasion and progression [22, 23].

In patients with GC, IL-10 secreted by tumor-associated macrophages regulates the proliferation and invasion of GC cells via c-mesenchymal epithelial transition protein/STAT3 signaling [24]. In GC, enrichment of intratumoral IL-10-producing macrophages is linked to poor clinical outcomes [25]. Furthermore, IL-10-producing B cells exert an antitumor effect by interacting with T cells. IL-10-producing B cells are reportedly enriched in the intratumoral environment, and significantly alter cytokine production by CD4 and CD8 T cells in GC [8]. B10 cells and plasmablasts suppress T cell-mediated pathogenic inflammation, as well as antiviral T cell responses, via IL-10 signaling [26]. B10 cells produce TGF-β to induce Foxp3 expression, thereby promoting the differentiation of Tregs [11].

In this study, the proportion of IL-10-producing B cells in the peripheral blood of patients with GC decreased after radical gastrectomy (Fig. 3A), and was higher in cancerous than normal mucosa (Fig. 4A). B10 cells prevent an effective antitumor immune response. In the presence and absence of cell death conditions, GC cell apoptosis and necrosis decreased 1 day after culture with IL-10 (Fig. 5A). However, GC cell proliferation was increased by IL-10 treatment (Fig. 5B). This data show direct preventing effect of IL-10 in antitumor response. Coculture of GC cells with patient-derived B cells significantly increased secretion of the inflammatory cytokines TNF-α, VEGF, IL-8, and IL-1β. IL-8 expression is correlated with a poor prognosis of GC because of its chemotactic, neutrophil-activating, and proangiogenetic effects. Our in vitro data, culture with IL-10 or co-culture with B cells, indicated that B10 cells likely promote GC cell proliferation by increasing the production of these proinflammatory cytokines [27]. In this study, there is a limitation that characteristics of the GC patients and healthy controls were not well matched because of the disease specificity. So, there is possibility of the differences in immune status due to age, sex, and comorbidities. However, our findings from in vitro study suggest that modulation of B10 could enhance antitumor effects, but further research and development is required for clinical application. It is difficult to directly target tumor cells, and enhancing systemic immunity does not exert an anticancer effect. Targeting B10 may improve the prognosis of GC as an adjuvant therapy that increases systemic and specific anticancer immunity to maximize the effects of surgery, chemotherapy, monoclonal antibodies, and immune checkpoint inhibitors.

## Conclusions

In conclusion, B10 cells may directly promote carcinogenesis by inducing local production of inflammatory mediators, such as IL-10, in GC. Our findings suggest that targeting B10 cells could improve the outcomes of antitumor immunotherapy.

## Data Availability

All data generated or analysed during this study are included in this published article and its supplementary information file.
